# Exploring the Relationship between Abnormal Communication Efficiency of Cerebral Cortex and Multiple Cognitive Functions in Mild Subcortical Stroke: A Resting-State fMRI Study

**DOI:** 10.3390/brainsci14080809

**Published:** 2024-08-12

**Authors:** Chang Liu, Jing Jing, Wanlin Zhu, Lijun Zuo

**Affiliations:** 1 Beijing Advanced Innovation Center for Biomedical Engineering, School of Biological Science and Medical Engineering, Beihang University, Beijing 100083, China; 2 China National Clinical Research Center for Neurological Diseases, Beijing 100070, China; jingj_bjttyy@163.com (J.J.); wanlin.zhu@ncrcnd.org.cn (W.Z.)

**Keywords:** post-stroke cognitive impairment, communication efficiency, neuroimaging, compensatory mechanism

## Abstract

Background: The purpose of this study was to explore the specific regions of abnormal cortical communication efficiency in patients with mild subcortical stroke and to investigate the relationship between these communication efficiency abnormalities and multidimensional cognition. Methods: The research involved 35 patients with mild strokes affecting the basal ganglia and 29 healthy controls (HC). Comprehensive neuroimaging and neuropsychological assessments were conducted. Stroke patients were categorized into post-stroke cognitive impairment (PSCI) (MoCA ≤ 22) and non-cognitively impaired stroke patients (NPSCI) (MoCA ≥ 23) based on their cognitive performance. Additionally, 22 patients were reassessed three months later. Results: PSCI patients, compared to HC and NPSCI groups, had significantly higher communication efficiency in specific brain regions. A notable finding was the significant correlation between increased communication efficiency in the medioventral occipital cortex and multidimensional cognitive decline. However, this increased communication efficiency in PSCI patients lessened during the three-month follow-up period. Conclusions: the heightened communication efficiency in the medio-ventral occipital cortex may represent a compensatory mechanism for cognitive impairment in PSCI patients, which undergoes adjustment three months after stroke.

## 1. Introduction

Subcortical stroke refers to a stroke occurring in various brain structures located beneath the cerebral cortex. Compared to cortical strokes, the symptoms of subcortical strokes may be less obvious, but they significantly impact cognitive functions in patients [1,2]. Commonly affected areas include the basal ganglia, thalamus, internal capsule, brainstem, and cerebellum [3]. These regions are intricately connected to the cerebral cortex and play crucial roles in regulating movement, sensation, emotions, and cognitive functions [4,5]. Damage to any of these subcortical structures can disrupt these connections, leading to various cognitive impairments such as attention deficits, memory loss, executive dysfunction, language problems, slower information processing, mathematical abilities, financial abilities, and visuospatial skills [6,7,8,9]. The cognitive impairments caused by subcortical strokes depend on the affected region, stroke severity, and the promptness of observing changes in brain function post-stroke [10]. Therefore, exploring the mechanisms of brain function changes and their relationship with different cognitive functions in subcortical stroke is essential. This understanding is crucial for the timely assessment and treatment of post-stroke cognitive impairments (PSCI) and for improving patient outcomes.

The development of neuroimaging technologies has provided important tools and methods for exploring the mechanisms of brain diseases [11]. Functional magnetic resonance imaging (fMRI), in particular, indirectly measures neural activity by detecting changes in blood-oxygen-level-dependent (BOLD) signals in brain regions [12]. fMRI technology, combined with graph theory analysis, is widely used to study the spatial and temporal patterns of brain functional activity. This approach is employed to investigate abnormalities in brain functional networks in conditions such as cognitive impairment, depression, anxiety, and other neurodegenerative diseases [13]. Research indicates that a decline in functional connectivity between the hippocampus and the inferior parietal lobule is associated with cognitive impairment in ischemic stroke patients [14]. Functional network characteristics in PSCI can predict long-term cognitive outcomes after stroke [15]. However, the association between cognitive impairments caused by subcortical strokes and alterations in cortical brain network mechanisms remains unclear.

In brain networks, nodes typically represent neurons, brain regions, or functional modules, while edges represent connections between these nodes (such as axonal projections or functional connections) [16]. The presence of communication pathways between nodes means that brain regions can exchange information via connected edges, which is crucial for the integration and distribution of functions in the brain, including working memory, attention regulation, and cognitive control. The shortest path length between nodes is an important indicator of communication efficiency: the fewer edges along a path, the shorter the time for information to transmit in the network, resulting in higher communication efficiency [17]. Research suggests that following brain injury, there is a short-term increase in node communication efficiency in brain regions, which represents a compensatory pattern post-injury [18,19]. However, the mechanisms underlying node transmission efficiency in the cortical regions of patients with cognitive impairments following subcortical strokes have not been fully elucidated. At the moment, the relationship between regional changes in node communication efficiency in the cortical areas and diverse cognitive impairments remains unexplored.

Therefore, in this study, we explored the cortical communication efficiency in patients with subcortical strokes based on resting-state fMRI data. We calculated the regional node shortest path lengths in the cortical areas of the brain and investigated the brain regions where abnormal node shortest path lengths were observed in PSCI. Furthermore, we analyzed the close relationship between node communication efficiency in these regions and multidimensional cognitive functions. Additionally, we examined the changes in node communication efficiency observed in subcortical stroke patients three months post-stroke. We hypothesized that following subcortical strokes, specific regions of the cortical brain would exhibit changes in node communication efficiency, which might be related to cognitive functions.

## 2. Materials and Methods

### 2.1. Participants

In this study, 35 patients experiencing their first-ever mild stroke in the basal ganglia region were included. These patients were diagnosed by neurologists following World Health Organization criteria, and their conditions were confirmed through brain computed tomography (CT) or MRI scans [20]. The inclusion criteria for the patients were: age between 35 and 65 years; presence of a mild stroke localized to the basal ganglia or encompassing both the basal ganglia and semi-central regions, with no prior history of stroke or transient ischemic attack (TIA). All stroke patients received treatment at the Department of Neurology, Beijing Tiantan Hospital, affiliated with Capital Medical University, between 1 December 2014 and 31 May 2016. Each participant underwent comprehensive MRI scans, which included T1, T2, diffusion-weighted imaging (DWI), fluid-attenuated inversion recovery (FLAIR), magnetic resonance angiography (MRA), and susceptibility-weighted imaging (SWI). Among the mild stroke patients, 22 completed follow-up assessments three months post-stroke. Additional assessments for all study participants took place between the 10th and 14th day following their hospital admission when their conditions were relatively stable. Participants were excluded if they exhibited stroke mimic symptoms (such as seizures or migraines), had literacy issues, had neurological diseases other than stroke, psychiatric disorders, acute cerebral infarction in cortical or other regions, or clear demyelination visible on fluid-attenuated inversion recovery (FLAIR) imaging and any major physical and mental conditions that may impede cognitive assessments. We recruited 29 healthy control subjects who were matched for age, sex, education level, and handedness. Criteria for healthy controls included the absence of neurological or psychiatric disorders and no evidence of significant demyelination or lacunar infarction on their CT or MRI scans. These healthy controls underwent the same imaging examinations as the stroke patients. This study received approval from the Ethics Committee of Beijing Tiantan Hospital, and informed consent was obtained from all participants.

### 2.2. Cognitive Assessment

Within 10 days post-stroke, well-trained neurologists conducted comprehensive cognitive assessments of the participants. The overall cognitive evaluation was performed using the Montreal Cognitive Assessment (MoCA) scale (MoCA-Beijing scale) [21]. Specific cognitive functions were assessed through various psychological tests: Memory was evaluated using the Auditory Verbal Learning Test (AVLT) and the Rey-Osterrieth Complex Figure Test-Delayed Recall (RCFT-DR) [22]; Visuomotor speed was measured with the Symbol Digit Modality Test (SDMT) [23]; Language abilities were assessed with the Animal Fluency Test (AFT) and a 30-item version of the Boston Naming Test (BNT) [24]; Attention and executive function was tested using the Chinese-modified versions of the Trail Making Test (TMT)-A and (TMT)-B, along with the Stroop Color-Word Test (CWT) [25]. Detailed descriptions of these assessments can be found in our prior publications. Based on MoCA scores, patients with mild stroke were classified into two groups: 20 patients with PSCI had MoCA scores of 22 or lower, while 15 non-cognitively impaired stroke patients (NPSCI) had MoCA scores of 23 or higher. When using the MoCA-Beijing version to assess cognitive impairment in Chinese patients with acute mild ischemic stroke, a cutoff score of 23 is optimal for distinguishing between patients with PSCI and NPSCI [26]. The 22 mild stroke patients completed neurocognitive assessments three months after the initial evaluation.

### 2.3. Image Acquisition

MRI scans for all participants were conducted on a Siemens 3.0 T Prisma MRI scanner (Siemens Healthcare, Erlangen, Germany) at the Functional Neuroimaging Department of the Beijing Neurosurgical Institute, Capital Medical University. The imaging was performed using a single-shot echo-planar imaging (EPI) sequence. For high-resolution structural imaging, a T1-weighted anatomic sequence was obtained with the following parameters: repetition time (TR) of 2300 ms, echo time (TE) of 2.3 ms, flip angle of 8°, voxel dimensions of 0.94 × 0.94 × 1 mm³, and an image matrix of 256 × 256 × 192. For fMRI scans, the parameters were a TR of 2500 ms, TE of 30 ms, flip angle of 90°, voxel size of 2.86 × 2.86 × 3 mm³, an image matrix of 70 × 70 × 43, and a total of 200 volumes.

### 2.4. Construction of Brain Functional Networks

#### 2.4.1. Image Preprocessing

The preprocessing of the fMRI data involved several crucial steps to ensure accuracy and reliability. First, the middle slice was selected as the reference for slice timing correction. Next, a six-parameter rigid-body spatial transformation was applied to realign the fMRI time series, mitigating the effects of head movements. Functional and structural images were then co-registered and normalized to the Montreal Neurological Institute (MNI) space, followed by resampling to 3-mm isotropic voxels. The data were smoothed using a Gaussian kernel with a 6-mm full width at half maximum. To further refine the data, detrending and band-pass filtering (0.01–0.08 Hz) were performed. To address fluctuations in the BOLD signal that are not related to neuronal activity, we regressed out several nuisance covariates: the global mean signal, the six parameters of head motion, the mean signal from cerebrospinal fluid, and the mean signal from white matter. This approach helped in minimizing the impact of motion-related and other non-neuronal variations on the fMRI data.

#### 2.4.2. Network Construction

To construct the functional network, we based our analysis on the strength values of functional connectivity. We utilized the Human Brainnetome atlas [27] to divide the brain into 246 distinct regions. Using this partitioning, we generated a functional connectivity map by calculating the Pearson correlation coefficients between each pair of brain regions. These correlation values were then converted into z-scores using the Fisher r-to-z transformation, which allowed us to create a whole-brain connectivity matrix for each participant. To ensure meaningful connectivity within the brain’s functional networks while reducing the likelihood of spurious correlations, we applied a sparsity threshold of 0.15 to the functional connectivity matrix. Additionally, the cerebral cortex was delineated into seven functional networks based on the Yeo atlas [28], including the visual, somatomotor, dorsal attention, ventral attention, limbic, frontoparietal, and default mode networks.

### 2.5. Node Communication Efficiency Analysis

In this study, we computed the node’s shortest path length for each node within the network to quantify the routing efficiency of each node relative to all other nodes. We calculated the shortest path lengths in the weighted network; the detailed steps are as follows: (1) we determined the shortest path length between each pair of nodes in the weighted network. The results were stored in a matrix *D*_1_, where *D*_1_(*i*,*j*) represents the shortest path length from node *i* to node *j*. (2) To address situations where the distance between a node and itself is zero (*i = j*), which could lead to division by zero errors in subsequent calculations. We constructed a diagonal matrix *M*, where the diagonal elements are set to infinity. We then added *M* to *D*_1_ and considered the resulting matrix for further computation.

(1) $$M_{\infty }(i,j)=\left\{\begin{array}{ll} \infty & \mathrm{if}i=j\\ 0 & \mathrm{if}i\neq j \end{array}\right.$$

(2) $$D_{2\;}=\frac{1}{D_{1\;}+M_{\infty }}$$

(3) For each node *i*, we calculated the average reciprocal of the shortest path lengths to all other nodes. Subsequently, we took the reciprocal of this average to obtain the average shortest path length for each node.

(3) $$\mathrm{Shortest}\;\mathrm{path}\;\mathrm{lengths}=\frac{N-1}{\sum_{j=1}^{N}\;D_{2}(i,j)}$$

**Figure 1:**
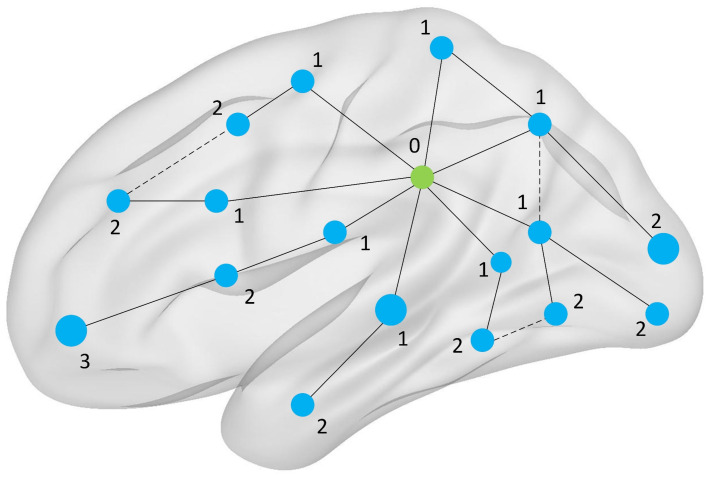
**Schematic Diagram of Node Shortest Path Lengths in Brain Network.** In a brain network, each node typically represents a specific brain region, and edges represent the connections between these nodes. The transmission path of a node is the sequence of edges that must be traversed to move from one node to another. Among all possible paths, the path between two nodes that pass through the fewest edges is called the “shortest path”. In our research, we use weighted brain functional networks. Here, the shortest path is recorded as the one with the minimum sum of edge weights, which represents the path with the least cumulative weight among those with the fewest edges.

For a detailed visualization of the process used to construct the node shortest path, please refer to Figure 1.

### 2.6. Statistical Analysis

To analyze the differences between groups in terms of clinical data, cognitive performance, and the coupling of brain structural and functional connectivity, we utilized various statistical methods based on the nature and distribution of the data. For continuous variables that followed a normal distribution, we reported the data as mean ± standard deviation. Comparisons between two groups were conducted using independent samples *t*-tests, while comparisons among three groups were performed with one-way analysis of variance (ANOVA). For continuous variables that did not follow a normal distribution, we presented the data as median (quartiles). The Wilcoxon test was used for comparisons between two groups, and the Kruskal-Wallis test was applied for comparisons among three groups. Categorical variables were expressed as percentages. To compare proportions between two or three groups, we used the chi-square test. To explore the relationship between cognitive scores and the brain node’s shortest path, we conducted multiple linear regression analyses. Age, gender, and education level were included as covariates to control for their potential confounding effects. Paired *t*-tests were used to evaluate changes in the brain node’s shortest path as well as cognitive performance from baseline to three months post-mild stroke. All statistical analyses were carried out using SPSS software version 26.0. All comparisons among the three groups were corrected using the Bonferroni method. Results were considered statistically significant if the *p*-value was less than 0.05.

## 3. Results

### 3.1. Demographics, Clinical and Neuropsychological Characteristics

The study involved a total of 35 patients experiencing their first mild stroke with infarction in the basal ganglia, who were assessed initially between 1 December 2014 and 31 May 2016. Follow-up assessments were conducted on 22 of these individuals three months later. At baseline, the study group included 20 patients with PSCI, 15 NPSCI, and 29 HC. The average age of the participants was around 50 years, with a majority being male. Detailed characteristics of the participants can be found in Table 1. Patients in the PSCI group, in comparison to the HC group, had higher cardiovascular risks and reported more frequent smoking and alcohol consumption.

For cognitive levels across the groups, please refer to Table 2. Patients in the PSCI group scored significantly lower than both the NPSCI and HC groups across multiple cognitive domains, including the MoCA score, memory, visuomotor speed, language, and attention/executive function (all with *p* ≤ 0.001). Additionally, compared to the HC group, the NPSCI group exhibited significantly lower performance in MoCA (*p* = 0.018), memory (*p* = 0.004), visuomotor speed (*p* < 0.001), and attention/executive function (*p* = 0.011).

### 3.2. Brain Node Shortest Path Comparison between Groups

**Figure 2:**
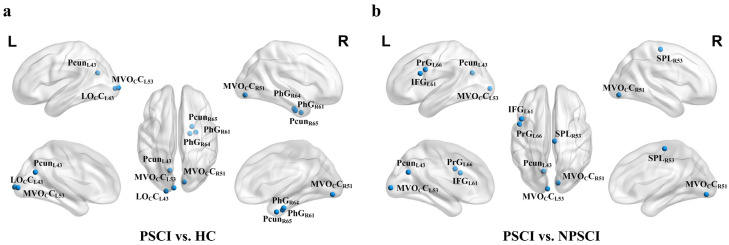
**Brain node shortest path lengths inter-group comparison graph.** (**a**) Brain regions exhibiting significant differences in node shortest path lengths between the PSCI and HC groups. (**b**) Brain regions exhibiting significant differences in node shortest path lengths between the PSCI and NPSCI groups. The blue indicates a decrease in brain node shortest path length values. Detailed information regarding brain regions is provided in Table 3 and Supplementary Table S1. HC, healthy controls; NPSCI, post-stroke patients without cognitive impairment; PSCI, post-stroke patients with cognitive impairment.

We compared brain nodes’ shortest path among the three groups: PSCI, NPSCI, and HC. ANOVA identified significant differences in the coupling coefficients of 14 brain regions across the groups. These regions are predominantly distributed in the limbic network and the visual network. To explore these differences in more detail, we conducted post-hoc *t*-tests on the node shortest path for these 14 regions, applying Bonferroni correction to account for multiple comparisons. The specific findings from these analyses are detailed in Figure 2 and Table 3. The result shows that the PSCI group exhibited a marked decrease in node shortest path compared to both the HC and NPSCI groups.

### 3.3. Brain Node Shortest Path and Cognition

**Figure 3:**
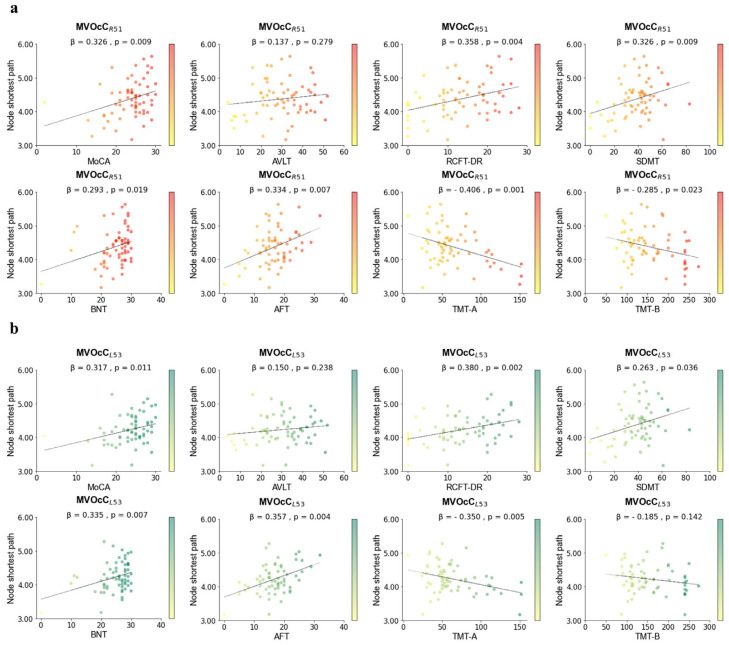
**Relationship between node shortest path length and multidimensional cognition in brain regionality.** (**a**). Relationship between node shortest path length and multidimensional cognition in MVOcC_R51_. (**b**). Relationship between node shortest path length and multidimensional cognition in MVOcC_L53_. Detailed information on brain regions is provided in Supplementary Table S1. MoCA, Montreal Cognitive Assessment Test; AVLT, Auditory verbal learning test; RCFT-DR, Rey-Osterrieth Complex Test-Delayed Recall; SMDT, Symbol Digit Modality Test; BNT, Boston Naming Test; AFT, Animal fluency test; TMT, Trail Making Test.

To explore the relationship between brain node shortest path lengths in the identified differential regions and cognitive function, we performed correlation analyses. We examined how the shortest path lengths in 14 distinct brain regions correlated with overall cognitive performance (MoCA score). Our analysis revealed that two specific brain regions (MVOcC_R51_ (beta = 0.326, *p* = 0.009) and MVOcC_L53_ (beta = 0.317, *p* = 0.011)) in medioventral occipital cortex displayed significant positive correlations with the MoCA score. We further explored how the shortest path lengths in these two regions related to various cognitive domains. Detailed findings can be found in Figure 3.

### 3.4. Brain Node Shortest Path Changes at Three-Month Follow-Up

At the three-month follow-up post-baseline, we analyzed the brain node shortest path lengths in two specific brain regions. Our findings indicated a significant increase in the shortest path length within the medioventral occipital cortex (MVOcC_R51_(t = 5.068, *p* < 0.001) and MVOcC_L53_ (t = 6.666, *p* < 0.001)).

## 4. Discussion

In this study, we investigated abnormal patterns of communication efficiency in cortical regions of the brain in patients with PSCI involving subcortical basal ganglia infarction. We observed that compared to HC and NPSCI patients, PSCI patients exhibited significantly increased communication efficiency in specific brain regions. Notably, the increased communication efficiency in the medio-ventral occipital cortex was correlated with cognitive decline, as measured by the MoCA total score and other diversified cognitive assessments. However, three months post-stroke, this trend of heightened communication efficiency showed a reduction in PSCI patients.

We employed multimodal neuroimaging and neuropsychological testing to evaluate stroke patients. Our findings indicated that patients with PSCI demonstrated poorer performance across all cognitive domains when compared to NPSCI and HC participants, with particularly pronounced deficits in visual and memory functions. Moreover, we observed region-specific increases in cortical communication efficiency in PSCI patients; these regions are mainly distributed in the edge network and the visual network. This finding indicates that, following a stroke, unaffected brain regions may form new connections or enhance existing ones. Studies suggest that after a stroke, increased functional connectivity in non-damaged regions may be related to axonal sprouting to establish new projection patterns and connections [29]. Our findings can be interpreted from a compensatory perspective, aligning with the theory that the brain compensates for damage by reorganizing and forming new connections among intact neurons [30], highlighting the brain’s remarkable ability to adapt to and mitigate the impact of localized injuries [31].

We conducted an in-depth analysis of how the communication efficiency at individual brain nodes relates to various cognitive functions in stroke patients. We have discovered an overall trend: lower cognitive levels are correlated with higher communication efficiency. This is a novel finding because post-stroke cognitive impairments typically involve brain network reorganization and functional damage, which can affect information processing efficiency [32]. Some studies suggest that post-stroke cognitive impairment may lead to decreased efficiency in certain parts or nodes of the brain network, resulting in reduced or delayed information transmission [33,34]. However, in specific brain regions, increased information efficiency may also be observed, possibly due to compensatory changes in the adaptive reorganization of the nervous system [35]. We found that increased communication efficiency in the medioventral occipital cortex was associated with multiple levels of cognitive decline. This finding extends previous research. The medioventral occipital cortex, located in the medial and ventral parts of the occipital lobe, plays a crucial role in visual processing, especially in higher-level visual functions [36,37]. Research has shown that the medioventral occipital cortex connects indirectly to the basal ganglia via the parietal and frontal cortices [38]. These cortical regions are responsible for processing higher-order visual information from the medioventral occipital cortex and, through their connections with the basal ganglia, play a critical role in regulating motor responses and decision-making processes [39]. In patients with basal ganglia stroke, the disruption or reduction in blood supply to nearby brain tissue can lead to neuronal damage or death, subsequently impairing cognitive functions [40,41]. In our study, we observed a significant decline in visual and memory functions in PSCI patients who experienced basal ganglia strokes. This intricate network enhanced communication efficiency in the medioventral occipital cortex could reflect an adaptive response to support cognitive functions affected by the stroke.

Our study found that three months after a stroke, the shortest path length in the medioventral occipital cortex region of PSCI patients significantly increased, indicating a weakening of the compensatory mode. The first three months following a stroke are generally considered the acute and subacute phases [36]. During this period, the brain’s compensatory mechanisms are particularly pronounced [42]. At the three-month mark, these mechanisms begin to stabilize, and the compensatory connections that have formed start to optimize, consolidate, and refine. As the brain gradually returns to normal function, there is a reduction in excessive compensatory activity [43]. Despite these changes, we did not observe significant cognitive improvements three months post-stroke. This lack of observable improvement may be attributed to the varying severity of cognitive impairment, leading to different recovery trajectories among patients [2]. The initial three months post-stroke often represent one of the most critical periods for cognitive dysfunction [44].

The strengths of this study lie in its comprehensive examination of the atypical patterns of cortical information transmission efficiency in PSCI and the changes in these patterns over the course of three months following a stroke. We also examined the relationship between cortical information transmission efficiency and multidimensional cognitive functions. Our findings provide a new perspective on the neural mechanisms underlying PSCI. However, we acknowledge several limitations in our research. First, the sample size for the follow-up period was relatively small. To validate the reliability of our results, we plan to include a larger cohort of stroke patients and increase the number of follow-up participants in future studies. Second, we recruited patients under the age of 65 to avoid the confounding effects of aging on cognition. Consequently, our study population has an average age lower than typical stroke patients, which suggests they may exhibit higher neuroplasticity and faster recovery rates compared to them. Lastly, due to difficulties in follow-up with stroke patients, only a subset of participants underwent neuroimaging and psychological assessments at three months. The reduced sample size could introduce bias to the follow-up results. In future studies, we plan to increase the sample size to ensure further accuracy of our findings.

## 5. Conclusions

This study provides a detailed regional analysis of cortical information transmission efficiency in PSCI patients with basal ganglia stroke and explores its relationship with multidimensional cognitive functions. We found notably efficient communication efficiency in the visual and default mode networks in PSCI. The increased regional transmission efficiency in the medioventral occipital cortex is associated with cognitive impairment. These findings suggest a compensatory reorganization of functional connections in the basal ganglia stroke region, which diminishes three months post-stroke. The research offers a novel perspective on understanding the neural mechanisms in PSCI patients with basal ganglia stroke and the mechanisms of change during cognitive rehabilitation.

## Figures and Tables

**Table 1:** Clinical and Demographic Characteristics.

	HC (*n* = 29)	PSCI (*n* = 20)	NPSCI (*n* = 15)	*p* Value	HC vs. NPSCI *p* Value	HC vs. PSCI *p* Value	NPSCI vs. PSCI *p* Value
Demographic information							
Age (years, mean ± SD)	51.07 ± 6.77	54.00 ± 9.41	48.00 ± 11.39	0.215	0.282	0.227	0.125
Male (%)	72.4	77.8	78.6	0.931	0.665	0.682	1
Education level (%) (Junior high school or below/ High school/University)	20.7/34.5/44.8	45/35/20	20/26.7/53.3	0.051	0.283	0.076	0.021
Vascular risk factors (%)							
Pathoglycemia	0	27.8	7.7	0.006	0.310	0.006	-
Diabetes	0	38.9	15.4	0.001	0.091	0.001	0.155
Hypertension	6.9	77.8	46.2	<0.001	0.003	<0.001	0.069
Hyperlipidemia	3.4	88.9	69.2	<0.001	<0.001	<0.001	0.208
Coronary ArteryDisease	0	11.1	0	0.131	-	0.142	0.497
Peripheral ArterialDisease	0	5.6	23.1	0.018	0.025	0.383	0.284
Intracranial Arterial Stenosis	0	11.1	0	0.131	-	0.142	0.497
Lifestyle factors (%)							
Current or ever smoking	10.3	72.2	61.5	<0.001	<0.001	<0.001	0.530
Current or ever drinking	13.8	61.1	76.9	<0.001	<0.001	0.001	0.353

Note: Data are presented as mean ± standard deviation (SD) or percentage number (%). Reported *p* values were obtained from independent *t* tests for age, education. All *p* values are corrected for multiple comparison using FDR correction. HC = healthy controls; NPSCI = post-stroke patients without cognitive impairment; PSCI= post-stroke patients with cognitive impairment.

**Table 2:** The Difference in Cognitive Function among Three Diagnostic Groups.

	HC (*n* = 29)	PSCI (*n* = 20)	NPSCI (*n* = 15)	*p* Value	HC vs. NPSCI *p* Value	HC vs. PSCI *p* Value	NPSCI vs. PSCI *p* Value
Global cognition							
MoCA2	26 (28.5–24.5)	20 (24–17)	25 (27–21)	<0.001	0.018	<0.001	0.009
Memory							
AVLT1	36.17 ± 8.56	22.68 ± 12.45	27.27 ± 10.66	<0.001	0.004	<0.001	0.193
RCFT-DR2	19 (22.5–13)	4 (9.8–0)	14 (20–12)	<0.001	0.149	<0.001	<0.001
Visuomotor speed							
SDMT2	46 (55–43)	22.5 (35.8–12.8)	37 (43–29)	<0.001	<0.001	<0.001	0.005
Language							
BNT2	28 (29–26)	21 (24.8–20)	27 (28–22)	<0.001	0.153	<0.001	0.034
AFT1	19.76 ± 5.01	12.68 ± 4.44	17.40 ± 3.60	<0.001	0.114	<0.001	0.005
Attention/executive function							
TMT-A2	50 (59.5–37.5)	82.5 (115.3–60.3)	53 (62–35)	<0.001	0.495	<0.001	0.006
TMT-B2	118 (143.5–97.5)	220 (240–165)	144 (187–112)	0.001	0.058	<0.001	0.040
CWT-A time2	65 (77.5–57.5)	104 (182–70.8)	78 (81–60)	0.001	0.087	0.001	0.016
CWT time2	50 (50–50)	48.5 (50–42.3)	50 (50–49)	<0.001	0.011	<0.001	0.057

Note: Data are presented as mean ± standard deviation (SD) or median (quartile). HC = healthy controls; NPSCI = post-stroke patients without cognitive impairment; PSCI = post-stroke patients with cognitive impairment. MoCA = Montreal Cognitive Assessment Test; AVLT: Auditory verbal learning test; RCFT-DR = Rey-Osterrieth Complex Test-Delayed Recall; SMDT = Symbol Digit Modality Test; BNT = Boston Naming Test; AFT: Animal fluency test; TMT = Trail Making Test; CWT = Color Word Test.1. *t*-tests between two groups; ANOVA between three groups; 2. Wilcoxon test between two groups; Kruskal-Wallis test between three groups.

**Table 3:** Abnormal Regions of Shortest Path Lengths in the Brain.

ID	Brain Region	MNI Coordinates			F	*p* Value	HC vs. NPSCI		HC vs. PSCI		NPSCI vs. PSCI	
		X	Y	Z			t	*p*	t	*p*	t	*p*
29	IFGL61	−46	13	24	3.410	0.039	−0.172	0.382	0.140	0.521	0.312	0.034
63	PrGL66	−49	5	30	3.406	0.040	−0.225	0.196	0.108	0.989	0.333	0.037
108	FuGR33	43	−49	−19	3.240	0.046	−0.160	1.000	0.325	0.184	0.485	0.056
110	PhGR61	28	−8	−33	5.263	0.008	0.581	0.042	0.595	0.019	0.014	1.000
115	PhGL64	−19	−12	−30	5.760	0.005	0.664	0.005	0.388	0.123	−0.276	0.635
116	PhGR64	19	−10	−30	4.929	0.010	0.574	0.042	0.554	0.029	−0.020	1.000
118	PhGR65	22	1	−36	5.245	0.008	0.359	0.278	0.614	0.007	0.254	0.795
130	SPLR53	5	−21	61	5.031	0.009	−0.357	0.016	0.013	1.000	0.370	0.021
151	PCunL43	−12	−67	25	5.385	0.007	−0.005	1.000	0.308	0.011	0.313	0.033
183	CGL75	−5	7	37	3.738	0.029	−0.326	0.042	0.003	1.000	0.329	0.062
184	CGR75	4	6	38	3.336	0.042	−0.336	0.042	−0.057	1.000	0.279	0.164
190	MVOcCR51	10	−85	−9	4.422	0.016	−0.158	1.000	0.339	0.033	0.497	0.020
193	MVOcCL53	−6	−94	1	4.905	0.011	−0.038	1.000	0.325	0.022	0.364	0.031
203	LOcCL43	−18	−99	2	3.712	0.030	0.010	1.000	0.288	0.039	0.277	0.120

Note: HC = healthy controls; NPSCI = post-stroke patients without cognitive impairment; PSCI = post-stroke patients with cognitive impairment; MNI = Montreal Neurological Institute. L = left; R = right. Detailed information on brain regions can be found in Supplementary Table S1.

## Data Availability

The data that support the findings of this study are available from the corresponding author upon request.

## Supplementary Materials

The following supporting information can be downloaded at https://www.mdpi.com/article/10.3390/brainsci14080809/s1, Supplementary Table S1: Details of the 246 regions in the Human Brainnetome atlas.
