# Supplementary material for: Exploring the Relationship between Abnormal Communication Efficiency of Cerebral Cortex and Multiple Cognitive Functions in Mild Subcortical Stroke: A Resting-State fMRI Study
Source: Brain Sci. 2024 Aug 12;14(8):809. doi: 10.3390/brainsci14080809 (PMC11352420; doi:10.3390/brainsci14080809)
Supplement: Supplementary file 1 [file brainsci-14-00809-s001.zip › brainsci-3110534-supplementary.pdf]

Supplementary Table S1 Details of the 246 regions in Human Brainnetome atlas

Lobe	Gyrus	Left and Right Hemisphere	Anatomical and modified Cyto-architectonic descriptions	lh.MNI(X,Y,Z)	rh.MNI(X,Y,Z)
Frontal Lobe	SFG, Superior Frontal Gyrus	SFG_L(R)_7_1	A8m, medial area 8	-5 ,15, 54	7, 16, 54
		SFG_L(R)_7_2	A8dl, dorsolateral area 8	-18, 24, 53	22, 26, 51
		SFG_L(R)_7_3	A9l, lateral area 9	-11, 49, 40	13, 48, 40
		SFG_L(R)_7_4	A6dl, dorsolateral area 6	-18, -1, 65	20, 4, 64
		SFG_L(R)_7_5	A6m, medial area 6	-6, -5, 58	7, -4, 60
		SFG_L(R)_7_6	A9m, medial area 9	-5, 36, 38	6, 38, 35
		SFG_L(R)_7_7	A10m, medial area 10	-8, 56, 15	8, 58, 13
	MFG, Middle Frontal Gyrus	MFG_L(R)_7_1	A9/46d, dorsal area 9/46	-27, 43, 31	30, 37, 36
		MFG_L(R)_7_2	IFJ, inferior frontal junction	-42, 13, 36	42, 11, 39
		MFG_L(R)_7_3	A46, area 46	-28, 56, 12	28, 55, 17
		MFG_L(R)_7_4	A9/46v, ventral area 9/46	-41, 41, 16	42, 44, 14
		MFG_L(R)_7_5	A8vl, ventrolateral area 8	-33, 23, 45	42, 27, 39
		MFG_L(R)_7_6	A6vl, ventrolateral area 6	-32, 4, 55	34, 8, 54
		MFG_L(R)_7_7	A10l, lateral area10	-26, 60, -6	25, 61, -4
	IFG, Inferior Frontal Gyrus	IFG_L(R)_6_1	A44d, dorsal area 44	-46, 13, 24	45, 16, 25
		IFG_L(R)_6_2	IFS, inferior frontal sulcus	-47, 32, 14	48, 35, 13
		IFG_L(R)_6_3	A45c, caudal area 45	-53, 23, 11	54, 24, 12
		IFG_L(R)_6_4	A45r, rostral area 45	-49, 36, -3	51, 36, -1
		IFG_L(R)_6_5	A44op, opercular area 44	-39, 23, 4	42, 22, 3
		IFG_L(R)_6_6	A44v, ventral area 44	-52, 13, 6	54, 14, 11
	OrG, Orbital Gyrus	OrG_L(R)_6_1	A14m, medial area 14	-7, 54, -7	6, 47, -7
		OrG_L(R)_6_2	A12/47o, orbital area 12/47	-36, 33, -16	40, 39, -14
		OrG_L(R)_6_3	A11l, lateral area 11	-23, 38, -18	23, 36, -18
		OrG_L(R)_6_4	A11m, medial area 11	-6, 52, -19	6, 57, -16
		OrG_L(R)_6_5	A13, area 13	-10, 18, -19	9, 20, -19
		OrG_L(R)_6_6	A12/47l, lateral area 12/47	-41, 32, -9	42, 31, -9
	PrG, Precentral Gyrus	PrG_L(R)_6_1	A4hf, area 4(head and face region)	-49, -8, 39	55, -2, 33
		PrG_L(R)_6_2	A6cdl, caudal dorsolateral area 6	-32, -9, 58	33, -7, 57
		PrG_L(R)_6_3	A4ul, area 4(upper limb region)	-26, -25, 63	34, -19, 59
		PrG_L(R)_6_4	A4t, area 4(trunk region)	-13, -20, 73	15, -22, 71
		PrG_L(R)_6_5	A4tl, area 4(tongue and larynx region)	-52, 0, 8	54, 4, 9
		PrG_L(R)_6_6	A6cvl, caudal ventrolateral area 6	-49, 5, 30	51, 7, 30
	PCL, Paracentral Lobule	PCL_L(R)_2_1	A1/2/3ll, area1/2/3 (lower limb region)	-8, -38, 58	10, -34, 54
		PCL_L(R)_2_2	A4ll, area 4, (lower limb region)	-4, -23, 61	5, -21, 61
Temporal Lobe	STG, Superior Temporal Gyrus	STG_L(R)_6_1	A38m, medial area 38	-32, 14, -34	31, 15, -34
		STG_L(R)_6_2	A41/42, area 41/42	-54, -32, 12	54, -24, 11
		STG_L(R)_6_3	TE1.0 and TE1.2	-50, -11, 1	51, -4, -1
		STG_L(R)_6_4	A22c, caudal area 22	-62, -33, 7	66, -20, 6
		STG_L(R)_6_5	A38l, lateral area 38	-45, 11, -20	47, 12, -20
		STG_L(R)_6_6	A22r, rostral area 22	-55, -3, -10	56, -12, -5

	MTG, Middle Temporal Gyrus	MTG_L(R)_4_1	A21c, caudal area 21	-65, -30, -12	65, -29, -13
		MTG_L(R)_4_2	A21r, rostral area 21	-53, 2, -30	51, 6, -32
		MTG_L(R)_4_3	A37dl, dorsolateral area37	-59, -58, 4	60, -53, 3
		MTG_L(R)_4_4	aSTS, anterior superior temporal sulcus	-58, -20, -9	58, -16, -10
	ITG, Inferior Temporal Gyrus	ITG_L(R)_7_1	A20iv, intermediate ventral area 20	-45, -26, -27	46, -14, -33
		ITG_L(R)_7_2	A37elv, extreme lateroventral area37	-51, -57, -15	53, -52, -18
		ITG_L(R)_7_3	A20r, rostral area 20	-43, -2, -41	40, 0, -43
		ITG_L(R)_7_4	A20il, intermediate lateral area 20	-56, -16, -28	55, -11, -32
		ITG_L(R)_7_5	A37vl, ventrolateral area 37	-55, -60, -6	54, -57, -8
		ITG_L(R)_7_6	A20cl, caudolateral of area 20	-59, -42, -16	61, -40, -17
		ITG_L(R)_7_7	A20cv, caudoventral of area 20	-55, -31, -27	54, -31, -26
	FuG, Fusiform Gyrus	FuG_L(R)_3_1	A20rv, rostroventral area 20	-33, -16, -32	33, -15, -34
		FuG_L(R)_3_2	A37mv, medioventral area37	-31, -64, -14	31, -62, -14
		FuG_L(R)_3_3	A37lv, lateroventral area37	-42, -51, -17	43, -49, -19
	PhG, Parahippocampal Gyrus	PhG_L(R)_6_1	A35/36r, rostral area 35/36	-27, -7, -34	28, -8, -33
		PhG_L(R)_6_2	A35/36c, caudal area 35/36	-25, -25, -26	26, -23, -27
		PhG_L(R)_6_3	TL, area TL (lateral PPHC, posterior parahippocampal gyrus)	-28, -32, -18	30, -30, -18
		PhG_L(R)_6_4	A28/34, area 28/34 (EC, entorhinal cortex)	-19, -12, -30	19, -10, -30
		PhG_L(R)_6_5	TI, area TI (temporal agranular insular cortex)	-23, 2, -32	22, 1, -36
		PhG_L(R)_6_6	TH, area TH (medial PPHC)	-17, -39, -10	19, -36, -11
	pSTS, posterior Superior Temporal Sulcus	pSTS_L(R)_2_1	rpSTS, rostroposterior superior temporal sulcus	-54, -40, 4	53, -37, 3
		pSTS_L(R)_2_2	cpSTS, caudoposterior superior temporal sulcus	-52, -50, 11	57, -40, 12
Parietal Lobe	SPL, Superior Parietal Lobule	SPL_L(R)_5_1	A7r, rostral area 7	-16, -60, 63	19, -57, 65
		SPL_L(R)_5_2	A7c, caudal area 7	-15, -71, 52	19, -69, 54
		SPL_L(R)_5_3	A5l, lateral area 5	-33, -47, 50	35, -42, 54
		SPL_L(R)_5_4	A7pc, postcentral area 7	-22, -47, 65	23, -43, 67
		SPL_L(R)_5_5	A7ip, intraparietal area 7(hIP3)	-27, -59, 54	31, -54, 53
	IPL, Inferior Parietal Lobule	IPL_L(R)_6_1	A39c, caudal area 39(PGp)	-34, -80, 29	45, -71, 20
		IPL_L(R)_6_2	A39rd, rostrodorsal area 39(Hip3)	-38, -61, 46	39, -65, 44
		IPL_L(R)_6_3	A40rd, rostrodorsal area 40(PFt)	-51, -33, 42	47, -35, 45
		IPL_L(R)_6_4	A40c, caudal area 40(PFm)	-56, -49, 38	57, -44, 38
		IPL_L(R)_6_5	A39rv, rostroventral area 39(PGa)	-47, -65, 26	53, -54, 25
		IPL_L(R)_6_6	A40rv, rostroventral area 40(PFop)	-53, -31, 23	55, -26, 26
	Pcun, Precuneus	PCun_L(R)_4_1	A7m, medial area 7(PEp)	-5, -63, 51	6, -65, 51
		PCun_L(R)_4_2	A5m, medial area 5(PEm)	-8, -47, 57	7, -47, 58
		PCun_L(R)_4_3	dmPOS, dorsomedial parietooccipital sulcus(PEr)	-12, -67, 25	16, -64, 25
		PCun_L(R)_4_4	A31, area 31 (Lc1)	-6, -55, 34	6, -54, 35
	PoG, Postcentral Gyrus	PoG_L(R)_4_1	A1/2/3ulhf, area 1/2/3(upper limb, head and face region)	-50, -16, 43	50, -14, 44
		PoG_L(R)_4_2	A1/2/3tonIa, area 1/2/3(tongue and larynx region)	-56, -14, 16	56, -10, 15
		PoG_L(R)_4_3	A2, area 2	-46, -30, 50	48, -24, 48
		PoG_L(R)_4_4	A1/2/3tru, area1/2/3(trunk region)	-21, -35, 68	20, -33, 69

Insular Lobe	INS, Insular Gyrus	INS_L(R)_6_1	G, hypergranular insula	-36, -20, 10	37, -18, 8
		INS_L(R)_6_2	vIa, ventral agranular insula	-32, 14, -13	33, 14, -13
		INS_L(R)_6_3	dIa, dorsal agranular insula	-34, 18, 1	36, 18, 1
		INS_L(R)_6_4	vId/vIg, ventral dysgranular and granular insula	-38, -4, -9	39, -2, -9
		INS_L(R)_6_5	dIg, dorsal granular insula	-38, -8, 8	39, -7, 8
		INS_L(R)_6_6	dId, dorsal dysgranular insula	-38, 5, 5	38, 5, 5
Limbic Lobe	CG, Cingulate Gyrus	CG_L(R)_7_1	A23d, dorsal area 23	-4, -39, 31	4, -37, 32
		CG_L(R)_7_2	A24rv, rostroventral area 24	-3, 8, 25	5, 22, 12
		CG_L(R)_7_3	A32p, pregenual area 32	-6, 34, 21	5, 28, 27
		CG_L(R)_7_4	A23v, ventral area 23	-8, -47, 10	9, -44, 11
		CG_L(R)_7_5	A24cd, caudodorsal area 24	-5, 7, 37	4, 6, 38
		CG_L(R)_7_6	A23c, caudal area 23	-7, -23, 41	6, -20, 40
		CG_L(R)_7_7	A32sg, subgenual area 32	-4, 39, -2	5, 41, 6
Occipital Lobe	MVOcC, MedioVentral Occipital Cortex	MVOcC _L(R)_5_1	cLinG, caudal lingual gyrus	-11, -82, -11	10, -85, -9
		MVOcC _L(R)_5_2	rCunG, rostral cuneus gyrus	-5, -81, 10	7, -76, 11
		MVOcC _L(R)_5_3	cCunG, caudal cuneus gyrus	-6, -94, 1	8, -90, 12
		MVOcC _L(R)_5_4	rLinG, rostral lingual gyrus	-17, -60, -6	18, -60, -7
		MVOcC _L(R)_5_5	vmPOS,ventromedial parietooccipital sulcus	-13, -68, 12	15, -63, 12
	LOcC, lateral Occipital Cortex	LOcC_L(R)_4_1	mOccG, middle occipital gyrus	-31, -89, 11	34, -86, 11
		LOcC _L(R)_4_2	V5/MT+, area V5/MT+	-46, -74, 3	48, -70, -1
		LOcC _L(R)_4_3	OPC, occipital polar cortex	-18, -99, 2	22, -97, 4
		LOcC_L(R)_4_4	iOccG, inferior occipital gyrus	-30, -88, -12	32, -85, -12
		LOcC _L(R)_2_1	msOccG, medial superior occipital gyrus	-11, -88, 31	16, -85, 34
		LOcC _L(R)_2_2	lsOccG, lateral superior occipital gyrus	-22, -77, 36	29, -75, 36
Subcortical Nuclei	Amyg, Amygdala	Amyg_L(R)_2_1	mAmyg, medial amygdala	-19, -2, -20	19, -2, -19
		Amyg_L(R)_2_2	lAmyg, lateral amygdala	-27, -4, -20	28, -3, -20
	Hipp, Hippocampus	Hipp_L(R)_2_1	rHipp, rostral hippocampus	-22, -14, -19	22, -12, -20
		Hipp_L(R)_2_2	cHipp, caudal hippocampus	-28, -30, -10	29, -27, -10
	BG, Basal Ganglia	BG_L(R)_6_1	vCa, ventral caudate	-12, 14, 0	15, 14, -2
		BG_L(R)_6_2	GP, globus pallidus	-22, -2, 4	22, -2, 3
		BG_L(R)_6_3	NAC, nucleus accumbens	-17, 3, -9	15, 8, -9
		BG_L(R)_6_4	vmPu, ventromedial putamen	-23, 7, -4	22, 8, -1
		BG_L(R)_6_5	dCa, dorsal caudate	-14, 2, 16	14, 5, 14
		BG_L(R)_6_6	dlPu, dorsolateral putamen	-28, -5, 2	29, -3, 1
	Tha, Thalamus	Tha_L(R)_8_1	mPFtha, medial pre-frontal thalamus	-7, -12, 5	7, -11, 6
		Tha_L(R)_8_2	mPMtha, pre-motor thalamus	-18, -13, 3	12, -14, 1
		Tha_L(R)_8_3	Stha, sensory thalamus	-18, -23, 4	18, -22, 3
		Tha_L(R)_8_4	rTtha, rostral temporal thalamus	-7, -14, 7	3, -13, 5
		Tha_L(R)_8_5	PPtha, posterior parietal thalamus	-16, -24, 6	15, -25, 6
		Tha_L(R)_8_6	Otha, occipital thalamus	-15, -28, 4	13, -27, 8
		Tha_L(R)_8_7	cTtha, caudal temporal thalamus	-12, -22, 13	10, -14, 14
		Tha_L(R)_8_8	lPFtha, lateral pre-frontal thalamus	-11, -14, 2	13, -16, 7
